# Chromosome microarray analysis combined with karyotype analysis is a powerful tool for the detection in pregnant women with high-risk indicators

**DOI:** 10.1186/s12884-023-06052-z

**Published:** 2023-11-11

**Authors:** Guanhua Qian, Liuyun Cai, Hong Yao, Xiaojing Dong

**Affiliations:** Obstetrics and Gynecology Department, The Second Affiliated Hospital of Chongqing Medical University, No. 74 Linjiang Road, Yuzhong District, Chongqing, 400010 China

**Keywords:** Prenatal diagnosis, Chromosomal microarray analysis (CMA), Copy number variation (CNV), Karyotype analysis

## Abstract

**Background:**

Karyotype analysis and fluorescence in situ hybridization (FISH) are commonly used for prenatal diagnosis, however they have many disadvantages. Chromosome microarray analysis (CMA) has the potential to overcome these disadvantages. This study aimed to evaluate the clinical value of CMA in the diagnosis of fetal chromosomal anomalies in southwest of China.

**Methods:**

A total of 3336 samples of amniotic fluid or umbilical cord blood from pregnant women with high-risk indicators at our center in southwest of China from June 2018 to January 2023 were included in the retrospective analysis. 3222 cases tested by CMA and karyotyping, 114 cases only tested by CMA.

**Results:**

3336 samples divided into 2911 cases with single and 425 cases with multiple high-risk indicators. The aneuploidy and pathogenic/likely pathogenic copy number variations (CNVs) of 2911 cases with single high-risk indicator were 4.43% (129/2911) and 2.44% (71/2911) respectively; the aneuploidy and pathogenic/likely pathogenic CNVs of 425 cases with multiple high-risk indicators were 6.82% (29/425) and 2.12% (9/425) respectively. The rate of aneuploidy increased significantly with pregnancy age or NT value. The detection rate of aneuploidy on cases with AMA combined NT ≥ 2.5 mm was significantly higher than that in cases only with AMA (*p* < 0.001); the detection rate of aneuploidy and pathogenic/likely pathogenic CNVs in cases with AMA combined NIPT high-risk were higher than that in cases only with AMA (*p* < 0.001, *p* < 0.05).

**Conclusions:**

The combined application of CMA and karyotyping were recommended in prenatal diagnosis for providing a scientific and accurate genetic diagnosis and improving the quality of prenatal genetic counseling.

## Background

Karyotype analysis, fluorescence in situ hybridization (FISH) and chromosome microarray analysis (CMA) are commonly used for invasive prenatal diagnosis based on the indicators, such as advanced maternal age (AMA), nuchal translucency (NT) ≥ 2.5 mm, abnormal result on maternal serum screening (MSS), high risk of non-invasive prenatal tests (NIPT), anomaly on ultrasonography (US), adverse pregnancy history (APH), parental genetic abnormalities, medication use or toxic exposure during pregnancy [1, 2].

Standard G-banded karyotype analysis is the conventional cytogenetic technique used in prenatal diagnosis, which can detect chromosomal aneuploidies, polyploidies, mosaic, and structural abnormalities, while it has several disadvantages such as low resolution and long turnaround time. The application of FISH is limited by the types of probes. The development of CMA allows us to detect micro deletions / duplication as low as 50–100 Kb [3]. It is a high-resolution and high-throughput molecular analysis technology for scanning the whole genome, which can detect chromosome polyploid, aneuploid, copy number variations (CNVs), uniparental diploid and mosaic. It has been recognized as a reasonable and effective tool for prenatal diagnosis and genetic counseling [4–6]. However, only using CMA along in prenatal diagnosis may lead to some limitations. As a result, we joint applied cytogenetic (karyotyping) and molecular genetic (CMA) techniques to analyze the prenatal diagnosis cases.

In this study, we retrospectively reviewed a cohort study of 3336 cases from southwest of China. Southwest of China is a mountainous and plateau region with a population of multiple ethnic groups. There are significant differences in terrain and population composition between the southwest and other regions of China. And there is a lack of corresponding research on prenatal diagnosis of chromosomal abnormalities. Therefore it is necessary to evaluate the clinical utility value of CMA and karyotype analysis in prenatal diagnosis, and explored the relationship between distribution of pathogenic/likely pathogenic chromosomal abnormalities and the high-risk indications of pregnancy in southwest of China.

## Methods

### Subjects

A total of 3336 samples including 3282 amniotic fluids and 54 umbilical cord bloods were collected at the Obstetrics and Gynecology prenatal diagnosis center of the Second Affiliated Hospital of Chongqing Medical University in southwest of China, from June 2018 to January 2023. All pregnant women have signed an informed consent form for the examination. The test was carried out after the hospital’s medical ethics committee’s approval.

In this study, AMA, NT ≥ 2.5 mm, abnormal result on MSS, high risk of NIPT, anomaly on US, APH and others (parental genetic abnormalities, medication use or toxic exposure during pregnancy abnormal) were indications for high-risk of pregnancy. The definition of AMA is 35 years or older. Ultrasound diagnoses physiological abnormalities in different organs of the fetus, including congenital heart disease, urinary system abnormalities, neurological abnormalities, craniofacial/cranial abnormalities, and other abnormalities.

### Karyotype analysis

Amniocentesis was performed to obtain the fetal sample after obtaining informed consent. Karyotype analysis was performed according to the standard protocol using G-banding at 450-band resolution [7].

### CMA

CMA is a whole genome chromosome variation detection technology with high-resolution. CMA is recommended as a first-line detection method for prenatal diagnosis of ultrasound abnormalities, fetal growth restriction, mental retardation, multiple malformations and other abnormalities [8]. According to different design of chip and detection principles, CMA can be divided into comparative genomic hybridization microarray (aCGH) and single nucleotide polymorphism microarray (SNP array). In particular, SNP array has many significant advantages in CNV analysis because it contains CNV and SNP dual high-resolution probes. In this study, SNP array was used to confirm the existence of the genomic variation in genomic DNA.

DNA was extracted from the cord blood or amniotic fluid cell using the QIAamp DNA Mini Kit (Qiagen, Hilden, Germany). The Infinium Global Screening Array (Illumina, San Diego, CA) comprised ~ 700 000 markers of SNP and CNV. The array was scanned with the iScan microarray scanning system (Illumina, San Diego, CA). Molecular karyotype analysis was performed by KaryoStudio 1.4.3.0 Build 37 software (Illumina, San Diego, CA). CNVs were classified and interpretation following the guidelines of the American College of Medical Genetics and Genomics (ACMGACMG)/ClinGen guideline: pathogenic, likely pathogenic, variants of uncertain significance (VUS), benign, likely benign [9, 10]. Benign and likely benign CNVs have little significance for prenatal diagnosis. In addition, fetuses with VUS generally do not have clinical phenotypes during the prenatal stage, so the statistical significance of VUS is not significant. Benign, likely benign and VUS CNVs cannot be used as a reference for clinic. Therefore, this study only counted the pathogenic and likely pathogenic CNVs.

### Bioinformatics

The chromosome regions were evaluated with the information provided by the Online Mendelian Inheritance in Man database (OMIM, http://omim.org/), the DECIPHER Database (http://decipher.sanger.ac.uk), UCSC database (http://genome.ucsc.edu), DGV database (http://dgv.tcag.ca/dgv/app/home) and ClinGen database (http://dosage.clinicalgenome.org/).

## Results

### Cases with single or multiple prenatal diagnostic indicators

Amniotic fluids or fetal umbilical cord bloods from 3336 cases with prenatal diagnostic indicators were successfully analyzed by CMA and karyotyping. 2911 cases only had a single prenatal diagnosis indicator: 1044 cases were AMA; 234 cases were NT ≥ 2.5 mm; 260 cases were abnormal result on MSS; 281 cases were high-risk of NIPT; 509 cases were anomaly on US; 318 cases were APH; 265 cases were other indicator (Table 1). Among 2911 cases with single indicator, 129 cases (129/2911, 4.43%) were aneuploidy; 30 cases (30/2911, 1.03%) were balanced chromosome structural rearrangements (or balanced chromosome abnormalities, BCAs); 71 cases (71/2916, 2.44%) were pathogenic and likely pathogenic CNVs (Table 1).

The remaining 425 cases had two high-risk indicators. Especially the group of AMA combined another high-risk indicator occupied a large portion, with a total of 340 cases. In 425 cases with multiple indicators, 29 cases (29/425, 6.82%) were aneuploidy; 8 cases (8/425, 1.88%) were BCAs; 9 cases (9/425, 2.12%) were pathogenic and likely pathogenic CNVs (Table 1).

**Table 1:** The indications for CMA and karotyping of 3336 cases with single and multiple high-risk indicators

Indications	Number	Aneuploidy		BCAs		CNV (P + LP)		CNV			
								P		LP	
AMA	1044	8	(0.77%)	8	(0.77%)	12	(1.15%)	9	(0.86%)	3	(0.29%)
NT(+)	234	21	(8.97%)	2	(0.85%)	3	(1.28%)	3	(1.28%)	0	(0.00%)
MSS(+)	260	2	(0.77%)	1	(0.38%)	7	(2.69%)	6	(2.31%)	1	(0.38%)
NIPT(+)	281	90	(32.03%)	0	(0.00%)	13	(4.63%)	12	(4.27%)	1	(0.36%)
US(+)	509	2	(0.39%)	3	(0.59%)	24	(4.72%)	19	(3.73%)	5	(0.98%)
APH	318	2	(0.63%)	2	(0.63%)	5	(1.57%)	4	(1.26%)	1	(0.31%)
Others	265	4	(1.51%)	14	(5.28%)	7	(2.64%)	3	(1.13%)	4	(1.51%)
AMA + NT(+)	51	6	(11.76%)	1	(1.96%)	1	(1.96%)	1	(1.96%)	0	(0.00%)
AMA + MSS(+)	2	0	(0.00%)	0	(0.00%)	0	(0.00%)	0	(0.00%)	0	(0.00%)
AMA + NIPT(+)	35	12	(34.29%)	0	(0.00%)	2	(5.71%)	0	(0.00%)	2	(5.71%)
AMA + US(+)	70	1	(1.43%)	0	(0.00%)	0	(0.00%)	0	(0.00%)	0	(0.00%)
AMA + APH	127	1	(0.79%)	2	(1.57%)	2	(1.57%)	2	(1.57%)	0	(0.00%)
AMA + Others	55	1	(1.82%)	3	(5.45%)	0	(0.00%)	0	(0.00%)	0	(0.00%)
NT(+) + NIPT(+)	2	2	(100.00%)	0	(0.00%)	0	(0.00%)	0	(0.00%)	0	(0.00%)
NT(+) + US(+)	10	1	(10.00%)	0	(0.00%)	1	(10.00%)	1	(10.00%)	0	(0.00%)
NT(+) + APH	6	0	(0.00%)	0	(0.00%)	1	(16.67%)	1	(16.67%)	0	(0.00%)
NT(+) + Others	3	0	(0.00%)	0	(0.00%)	0	(0.00%)	0	(0.00%)	0	(0.00%)
MSS(+) + US(+)	3	0	(0.00%)	0	(0.00%)	0	(0.00%)	0	(0.00%)	0	(0.00%)
MSS(+) + APH	7	0	(0.00%)	1	(14.29%)	0	(0.00%)	0	(0.00%)	0	(0.00%)
MSS(+) + Others	5	0	(0.00%)	0	(0.00%)	0	(0.00%)	0	(0.00%)	0	(0.00%)
NIPT(+) + US(+)	5	4	(80.00%)	0	(0.00%)	0	(0.00%)	0	(0.00%)	0	(0.00%)
NIPT(+) + Others	2	1	(50.00%)	0	(0.00%)	0	(0.00%)	0	(0.00%)	0	(0.00%)
US(+) + APH	14	0	(0.00%)	0	(0.00%)	0	(0.00%)	0	(0.00%)	0	(0.00%)
US(+) + Others	3	0	(0.00%)	0	(0.00%)	0	(0.00%)	0	(0.00%)	0	(0.00%)
APH + Others	25	0	(0.00%)	1	(4.00%)	2	(8.00%)	2	(8.00%)	0	(0.00%)
All	3336	158	(4.74%)	38	(1.14%)	80	(2.40%)	63	(1.89%)	17	(0.51%)

AMA: Advanced maternal age (≥ 35); NT(+):NT ≥ 2.5 mm; MSS(+): Abnormal result on maternal serum screening; NIPT(+): high-risk of NIPT; US(+): Anomaly on ultrasonograph; APH: Adverse pregnancy history; Others indicated parental genetic abnormalities, medication use or toxic exposure during pregnancy.

BCAs: balanced chromosome abnormalities; P: pathogenic; LP: likely pathogenic.

Aneuploidy included mosaic.

### Cases with independent AMA or AMA accompanied by another indicator

**Fig. 1:**
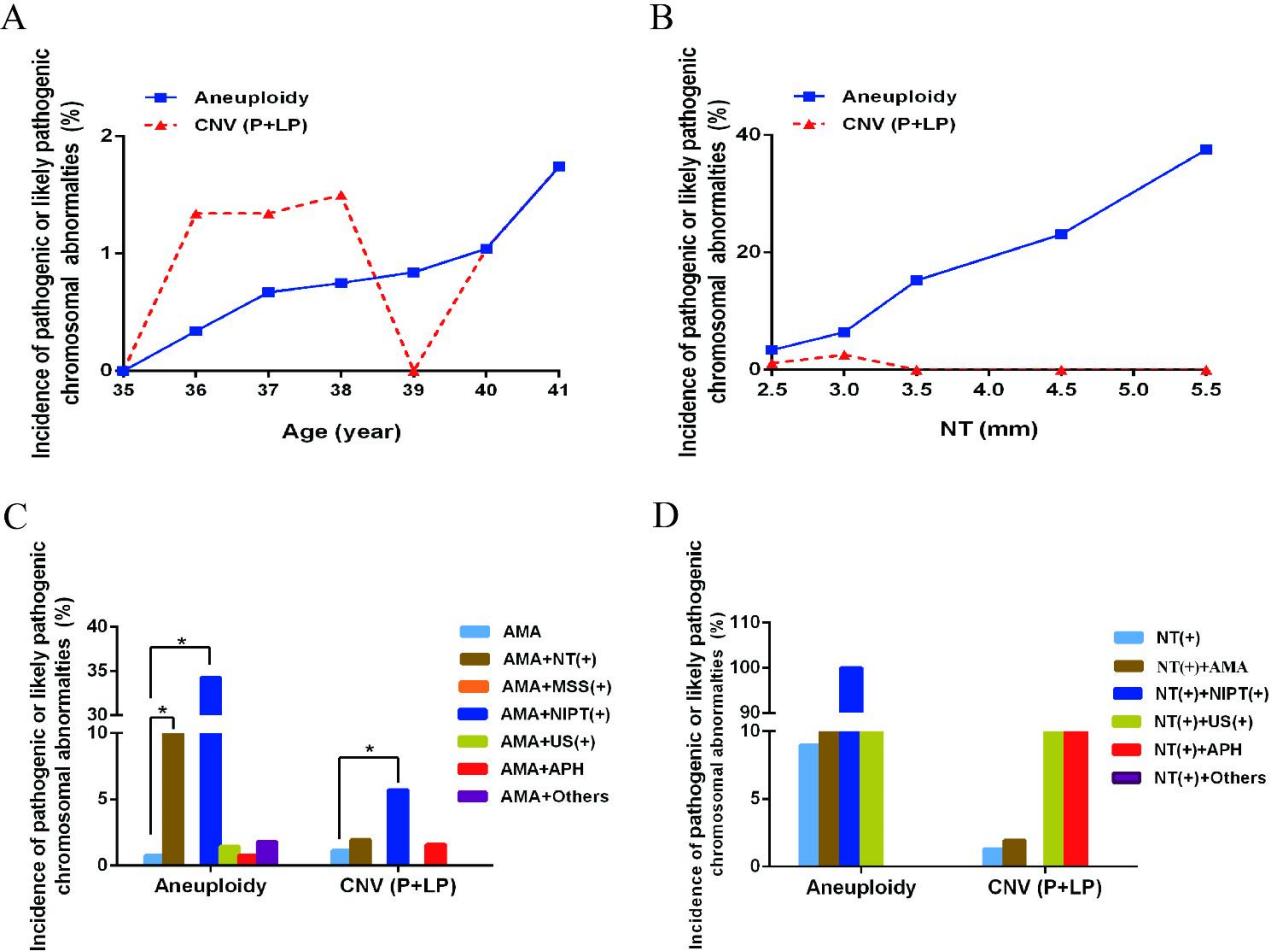
The incidences of pathogenic and likely pathogenic chromosomal aberrations in pregnancies of AMA or NT ≥ 2.5 mm. (A) The relationship between age and pathogenic/likely pathogenic chromosomal abnormality rate; (B) The relationship between NT value and pathogenic/likely pathogenic chromosomal abnormality rate; (C) Pathogenic/likely pathogenic chromosomal abnormality rate in cases of single AMA or AMA combined another indicators; (D) Pathogenic/likely pathogenic chromosomal abnormality rate in cases of single NT ≥ 2.5 mm or NT ≥ 2.5 mm combined another indicators. *p<0.05

A total of 1044 cases with single AMA performed CMA and karyotype analysis. 1044 samples were divided into seven subgroups according to the maternal age (35, 36, 37, 38, 39, 40, ≥ 41). Aneuploidy rate increased as pregnancy age increased, while pathogenic or likely pathogenic CNVs were not related to pregnancy age (Table 2; Fig. 1A). The results indicated that 20 samples were pathogenic or likely pathogenic chromosome abnormalities, including 8 (8/1044, 0.77%) cases of aneuploidy and 12 (12/1044, 1.15%) case of pathogenic or likely pathogenic CNVs (Table 2).

In 340 cases of AMA accompanied by another indicator, 51 cases were AMA combined NT (≥ 2.5 mm), 2 cases were AMA combined MSS abnormal result, 35 cases were AMA combined NIPT high-risk, 70 cases were AMA combined US anomaly, 127 cases were AMA combined APH and 55 cases were AMA combined others (Table 1). The results revealed that 26 samples were pathogenic or likely pathogenic chromosome abnormalities, including 21 (21/340, 6.18%) case of aneuploidy and 5 (5/340, 1.47%) case of pathogenic or likely pathogenic CNVs (Table 1; Fig. 1C). The detection rate of aneuploidy in group AMA combined NT ≥ 2.5 mm was significantly higher than that in group AMA (*p* < 0.001); the detection rate of aneuploidy and pathogenic/likely pathogenic CNVs in the group of AMA combined NIPT high-risk were higher than that in the group of AMA (*p* < 0.001, *p* = 0.027) (Fig. 1C).

**Table 2:** The results of CMA and karotyping on 1044 pregnancies with single advanced maternal age (≥ 35)

Age	Number	Aneuploidy		BCAs		CNV		CNV			
								P		LP	
35	77	0	(0.00%)	2	(2.60%)	0	(0.00%)	0	(0.00%)	0	(0.00%)
36	298	1	(0.34%)	4	(1.34%)	4	(1.34%)	2	(0.67%)	2	(0.67%)
37	149	1	(0.67%)	0	(0.00%)	2	(1.34%)	2	(1.34%)	0	(0.00%)
38	133	1	(0.75%)	1	(0.75%)	2	(1.50%)	2	(1.50%)	0	(0.00%)
39	119	1	(0.84%)	1	(0.84%)	0	(0.00%)	0	(0.00%)	0	(0.00%)
40	96	1	(1.04%)	0	(0.00%)	1	(1.04%)	1	(1.04%)	0	(0.00%)
≥ 41	172	3	(1.74%)	0	(0.00%)	3	(1.74%)	2	(1.16%)	1	(0.58%)
All	1044	8	(0.77%)	8	(0.77%)	12	(1.15%)	9	(0.86%)	3	(0.29%)

BCAs: balanced chromosome abnormalities; P: pathogenic; LP: likely pathogenic.

Aneuploidy included mosaic.

### Cases with independent NT ≥ 2.5 mm or NT ≥ 2.5 mm accompanied by another indicator

A total of 234 pregnancies were single NT (≥ 2.5 mm). The cases were divided into five types according to the specific value of NT (2.5–2.9, 3.0-3.4, 3.5–4.4, 4.5–5.4, ≥ 5.5 mm). The relationship between NT value and pathogenic or likely pathogenic chromosome abnormality rate was analyzed (Table 3; Fig. 1B). The result revealed that aneuploidy rate increased as NT value increased, while pathogenic or likely pathogenic CNVs were not related to NT value (Table 3; Fig. 1B).The data declared that 24 cases were pathogenic or likely pathogenic chromosome abnormalities, including 21 (21/234, 8.97%) case of aneuploidy and 3 (3/234, 1.28%) case of pathogenic or likely pathogenic CNVs (Table 3).

**Table 3:** The results of CMA and karotyping on 234 cases with single NT ≥ 2.5 mm

NT (mm)	Number	Aneuploidy		BCAs		CNV		CNV			
								P		LP	
2.5–2.9	89	3	(3.37%)	0	(0.00%)	1	(1.12%)	1	(1.12%)	0	(0.00%)
3.0-3.4	78	5	(6.41%)	1	(1.28%)	2	(2.56%)	2	(2.56%)	0	(0.00%)
3.5–4.4	46	7	(15.22%)	0	(0.00%)	0	(0.00%)	0	(0.00%)	0	(0.00%)
4.5–5.4	13	3	(23.08%)	1	(7.69%)	0	(0.00%)	0	(0.00%)	0	(0.00%)
≥ 5.5	8	3	(37.50%)	0	(0.00%)	0	(0.00%)	0	(0.00%)	0	(0.00%)
All	234	21	(8.97%)	2	(0.85%)	3	(1.28%)	3	(1.28%)	0	(0.00%)

BCAs: balanced chromosome abnormalities; P: pathogenic; LP: likely pathogenic.

Aneuploidy included mosaic

In 72 cases of NT ≥ 2.5 mm accompanied by another indicator, 51 cases were NT ≥ 2.5 mm combined AMA, 2 cases were NT ≥ 2.5 mm combined high-risk of NIPT, 10 cases were NT ≥ 2.5 mm combined US anomaly, 6 cases were NT ≥ 2.5 mm combined APH and 3 cases were NT ≥ 2.5 mm combined others (Table 1). The results suggested that 12 (12/72, 16.67%) cases were pathogenic or likely pathogenic chromosome abnormalities, including 9 (9/72, 12.50%) cases were aneuploidy and 3 cases (3/72, 4.17%) were pathogenic and likely pathogenic CNVs (Table 1; Fig. 1D ).

### Cases with independent high-risk of NIPT or accompanied by another indicator

281 cases with single high-risk of NIPT were identified in this research. The high-risk of NIPT were divided into five types, chromosome 13 (Chr 13), chromosome 18 (Chr 18), chromosome 21 (Chr 21), sex chromosomes (Chr sex) and other chromosomes (Chr other) except Chr 13, Chr 18, Chr 21 and Chr sex. CMA and karyotype analysis were performed to verify the abnormal NIPT results. 25 cases were NIPT abnormal on Chr 13, including 2 confirmed cases of trisomy 13; 29 cases were NIPT abnormal on Chr 18, including 9 confirmed cases of trisomy 18 aneuploidy and 1 confirmed case of mosaic; 44 cases were NIPT abnormal on Chr 21, including 27 confirmed cases of trisomy 21 and 3 confirmed case of mosaic, and 2 cases of pathogenic or likely pathogenic CNVs; 100 cases were NIPT abnormal on Chr sex, including 40 confirmed cases of aneuploidy 6 confirmed case of mosaic, and 4 cases of pathogenic CNVs; 83 cases were NIPT abnormal on Chr other, including 2 confirmed cases of mosaic aneuploidy and 7 cases of pathogenic or likely pathogenic CNVs (Table 4). The false positive rate and the false negative rate of abnormal NIPT were 63.35% and 0%.

44 cases were abnormal NIPT result accompanied by another indicator. Among them, the group of NIPT high-risk combined NT **≥** 2.5 mm and the group of NIPT high-risk combined US anomaly had the highest accuracy, reaching 100% (2/2) and 80% (4/5) respectively (Table 1).

**Table 4:** The results of CMA and karyotyping on 281 cases with single NIPT high-risk

NIPT	Number	Aneuploidy		BCAs		CNV		CNV			
								P		LP	
Chromosomes 13	25	2	(8.00%)	0	(0.00%)	0	(0.00%)	0	(0.00%)	0	(0.00%)
Chromosomes 18	29	10	(34.48%)	0	(0.00%)	0	(0.00%)	0	(0.00%)	0	(0.00%)
Chromosomes 21	44	30	(68.18%)	0	(0.00%)	2	(4.55%)	1	(2.27%)	1	(2.27%)
Sex chromosomes	100	46	(46.00%)	0	(0.00%)	4	(4.00%)	4	(4.00%)	0	(0.00%)
Other chromosomes	83	2	(2.41%)	0	(0.00%)	7	(8.43%)	7	(8.43%)	0	(0.00%)
All	281	90	(32.03%)	0	(0.00%)	13	(4.63%)	12	(4.27%)	1	(0.36%)

BCAs: balanced chromosome abnormalities; P: pathogenic; LP: likely pathogenic.

Other chromosomes indicate chromosomes except chr21, chr13, chr18 and sex chromosomes.

Aneuploidy included mosaic.

### Discrepancy between CMA and karyotype analysis

Among the 3336 cases studied in this study, 3222 underwent both CMA and karyotype analysis simultaneously, while 114 cases only underwent CMA due to 110 cases of gestational age > 26 weeks and 4 cases of amniotic fluid cell culture failed. Among the 3222 samples, 148 samples of common aneuploidy and 10 samples of mosaic aneuploidy were detected by CMA; while 144 samples of common aneuploidy, 4 samples of translocation aneuploidy and 13 samples of mosaic aneuploidy were detected by karyotype analysis. It should be noted that the 3 mosaic aneuploidy cases of the mosaic rate ≤ 20% have not been detected by CMA. In addition, 38 samples of BCAs were verified by karyotype analysis (Table 5). It’s purely coincidental that all pathogenic and likely pathogenic CNVs in this study appeared in the samples only undergoing CMA.

**Table 5:** Discrepancy between CMA and karyotype analysis

Methods	Anuiploidy			CNVs (P + LP)	BCAs
	common	translocatrion	mosaic		
CMA	148	0	10	0	0
Karyotype	144	4	13	0	38

BCAs: balanced chromosome abnormalities; P: pathogenic; LP: likely pathogenic.

## Discussion

In previous researches, there are many studies on CMA detection rate of cases with single high-risk indicator; while there are few studies on cases with multiple indicators. Moreover southwest of China has a unique geographical location, with many mountains and plateaus, making it a multi-ethnic region that differs from other populations in China. The corresponding research on the relationship between prenatal diagnosis of chromosomal abnormalities and pregnancies with high-risk indicators is lacked. Therefore, it is necessary to conduct relevant research on it.

In this study, 3336 cases from southwest of China were divided into two types, single high-risk indicator and multiple high-risk indicators. For cases with single high-risk indicator, the detection rate of pathogenic and likely pathogenic CNVs by CMA was 2.44%. The cases with multiple high-risk indicators had a higher risk on aneuploidy compared to cases with single high-risk indicator. However the risk of pathogenic and likely pathogenic was not changed. Especially, in cases with NT ≥ 2.5 mm merged NIPT high-risk, the prediction rate for aneuploidy was almost 100%, which was consistent with the results of prenatal diagnosis. Due to the limited number of cases with NT ≥ 2.5 mm merged NIPT high-risk in our study; further research is needed in the future.

In 1044 cases with single AMA, aneuploidy rate was 0.77% (8/1044), pathogenic and likely pathogenic CNVs was 1.15% (12/1044) (Table 2). The results verified that the rate of aneuploidy increased significantly as pregnancy age increased, while there was no such trend in the rate of pathogenic and likely pathogenic CNVs, which was consistent with previous studies [11, 12]. In 234 cases with single NT ≥ 2.5 mm, aneuploidy rate was 8.97% (21/234), pathogenic/likely pathogenic CNVs was 1.28% (3/234) (Table 3). Through the results, we identified the aneuploidy rate was closely related to the NT value, suggesting that it is extremely necessary to take NT as an important subject of prenatal screening [13, 14].

NIPT has been widely used for detecting fetal chromosome trisomy 13, 18, 21 and sex chromosome abnormality [15–17]. The positive rate of NIPT is approximately 1–2% in southern China and in Japan [18, 19]. The performance of NIPT for screening other fetal chromosome aneuploidies and CNVs is still limited. In this study, NIPT high-risk comprised of aneuploidies and CNVs of all autosomes and sex chromosomes. For Chr 13, Chr18 and Chr21, the accuracy levels of NIPT were 7.14%, 40.54% and 73.21% which suggesting that NIPT performed well in detecting Trisomy 21, but insufficient in Trisomy 13 and Trisomy 18. The accuracy rate of sex chromosomes aneuploidies on NIPT was 46.23%, while the accuracy rate of NIPT for detecting other chromosomes (except 13,18,21 and sex chromosomes) were poor, which was consistent with previous reports [18, 20]. This suggested that NIPT could only be used as a screening item, and interventional prenatal diagnosis must be performed for NIPT-positive pregnant.

Prenatal CMA is important for diagnosis of chromosomal abnormality. Compared to karyotyping, CMA does not require cells to be cultured, and has the advantages of high throughput, high resolution and high accuracy. It overcomes the disadvantages of karyotyping, e.g. cells culture failure, long time of culture cycle, incapable of micro deletions/duplications. However compared to CMA, karyotyping are more intuitive, and can detect BCAs and low proportion mosaic aneuploidy. In this study, 3 aneuploidy samples of the mosaic rate ≤ 20% and 38 samples of BCAs were only detected by karyotype analysis (Table 5). Therefor CMA and karyotyping should be used simultaneously in prenatal diagnosis to overcome these limitations and to provide a scientific and accurate genetic diagnosis for targeted improving the quality of prenatal genetic counseling and reduce the incidence of birth defects.

## Conclusions

In summary, a retrospective analysis was performed on a cohort of 3336 cases with high-risk indicators. The detection rate of aneuploidy and pathogenic/likely pathogenic CNVs by CMA and karyotyping was 4.83% (161/3336) and 2.40% (80/3336). 38 cases with BCAs were detected by karyotyping, which cannot identified by CMA. The aneuploidy rate of cases with multiple high-risk indicators or AMA was higher than that of the cases with single high-risk indicator or AMA, but pathogenic/likely pathogenic CNVs rate was not changed. CMA cannot be omitted for non-AMA pregnancy with single high-risk indicator. The combined application of CMA and karyotyping were recommended in prenatal diagnosis for providing a scientific and accurate genetic diagnosis and improving the quality of prenatal genetic counseling.

## Data Availability

The datasets used and/or analyzed during the current study are not publicly available because they contain the patients’ personal information, but are available from the corresponding author on reasonable request.

## Abbreviations

FISH: Fluorescence in situ hybridization
CMA: Chromosome microarray analysis
AMA: Advanced maternal age
NT: Nuchal translucency
MSS: Maternal serum screening
NIPT: Non-invasive prenatal tests
CNVs: Copy number variations
SNP: Single nucleotide polymorphism microarray
BCAs: Balanced chromosome abnormalities
P: Pathogenic
LP: Likely pathogenic
